# The cell biology of primary cell walls during salt stress

**DOI:** 10.1093/plcell/koac292

**Published:** 2022-09-23

**Authors:** Leia Colin, Felix Ruhnow, Jian-Kang Zhu, Chunzhao Zhao, Yang Zhao, Staffan Persson

**Affiliations:** Department of Plant and Environmental Sciences, University of Copenhagen, 1871 Frederiksberg C, Denmark; Department of Plant and Environmental Sciences, University of Copenhagen, 1871 Frederiksberg C, Denmark; School of Life Sciences, Institute of Advanced Biotechnology, Southern University of Science and Technology, Shenzhen 518055, China; Shanghai Center for Plant Stress Biology, CAS Center for Excellence in Molecular Plant Sciences, Chinese Academy of Sciences, Shanghai 200032, China; Shanghai Center for Plant Stress Biology, CAS Center for Excellence in Molecular Plant Sciences, Chinese Academy of Sciences, Shanghai 200032, China; Department of Plant and Environmental Sciences, University of Copenhagen, 1871 Frederiksberg C, Denmark; Copenhagen Plant Science Center, University of Copenhagen, 1871 Frederiksberg C, Denmark; Joint International Research Laboratory of Metabolic and Developmental Sciences, School of Life Sciences and Biotechnology, Shanghai Jiao Tong University, Minhang 200240, Shanghai, China

## Abstract

Salt stress simultaneously causes ionic toxicity, osmotic stress, and oxidative stress, which directly impact plant growth and development. Plants have developed numerous strategies to adapt to saline environments. Whereas some of these strategies have been investigated and exploited for crop improvement, much remains to be understood, including how salt stress is perceived by plants and how plants coordinate effective responses to the stress. It is, however, clear that the plant cell wall is the first contact point between external salt and the plant. In this context, significant advances in our understanding of halotropism, cell wall synthesis, and integrity surveillance, as well as salt-related cytoskeletal rearrangements, have been achieved. Indeed, molecular mechanisms underpinning some of these processes have recently been elucidated. In this review, we aim to provide insights into how plants respond and adapt to salt stress, with a special focus on primary cell wall biology in the model plant *Arabidopsis thaliana*.

## Teaser

This review examines the relationship between the plant cell wall and salt stress.

## Introduction

It is estimated that soil salinization affects more than 20% of all cultivated land, and approximately half of all irrigated land, in the world (Botella et al., 2007; Shrivastava and Kumar, 2015). Soil salinity severely threatens the entire life cycle of plants, in particular during seed germination, seedling establishment, and reproduction stages, leading to crop yield losses. Indeed, in 2021, the Food and Agriculture Organization of the United Nations estimated the global annual cost of salt-induced land degradation in irrigated areas to be US$27.3 billion related to lost crop productivity. Therefore, salt tolerance is increasingly considered an important agronomic trait for the breeding of modern crops. The tolerance of a plant to high salinity is determined by its capacity to control ion transport, to adjust reactive oxygen species (ROS) metabolism, turgor, and stomatal dynamics, as well as phenotypic plasticity. Increasing evidence indicates that cell wall integrity is important for plants to adapt to salt stress, as disruption of genes required for cell wall sensing or cell wall biosynthesis results in growth defects and reduced plant survival rate under salt stress (Endler et al., 2015; Zhao et al., 2019). In addition, cell wall anisotropy regulates directional growth and is thus a pre-requisite for plants to direct their growth away from patches of high salinity. In this review, we highlight recent cell biological aspects of salt stress in the context of cell wall biology, with a particular focus on primary cell wall in the model plant *Arabidopsis thaliana* (Arabidopsis).

## The plant cell wall

The plant cell wall is an extracellular matrix that encases virtually all plant cells. This structure supports many aspects of plant growth and development. Indeed, the cell wall is in many cases a strong but pliable material constantly remodeled to direct cell expansion (Cosgrove, 2005). From a biochemical standpoint, the plant cell wall can be considered as a hybrid material, consisting of load-bearing cellulose microfibrils (Cosgrove, 2005; Anderson and Kieber, 2020) embedded in a hydrated matrix of polysaccharides (hemicelluloses and pectins) and structural proteins. The relative amounts of these components, and the exact biochemical make-up of them, may vary not only from one species to another, but also within the same species, across organs and tissues, and even between neighboring cells in the same tissue. This diversity often confounds our knowledge about how cell wall biology contributes to plant growth and stress responses. Nevertheless, plant cell walls are typically classified into at least two types: primary and secondary cell walls. Primary walls are highly heterogeneous structures that are constantly remodeled to adapt to environmental and developmental cues, whereas secondary walls are strong and static structures that provide strength to support plant architecture and water transport through it. In this review, we mainly focus on salt stress in the context of cellulose in primary cell walls and refer the reader to other reviews on this topic relating to other wall types and polysaccharides (e.g. Chun et al., 2019; Getahun et al., 2019).

Primary cell walls not only support directed cell growth, but also provide a physical barrier that separates the cell surface from the environment and thus protects cells against environmental changes. Indeed, as sessile organisms, plants have to face a wide range of stresses, including various abiotic stresses that are detrimental to growth and development. The cell wall integrity (CWI) surveillance system (i.e. the surveillance of the cell wall status) plays a key role in stress perception and stress responses in plants (Novaković et al., 2018; Vaahtera et al., 2019; Rui and Dinneny, 2020). Although many questions remain to be addressed in this process, several wall sensors involved in CWI monitoring have been identified (Wolf, 2017). A number of these sensors belong to a large family of cell wall associated- and plasma membrane-localized *Catharanthus roseus* receptor-like kinase 1-like (CrRLK1L) proteins, which includes 17 members in Arabidopsis (Lindner et al., 2012). These sensors contain a transmembrane domain, and typically display a highly conserved cytoplasmic serine/threonine kinase domain, as well as an extracellular domain of ∼400 amino acids that varies in length and sequence (Wolf, 2017). This extracellular domain is proposed to bind to cell wall components, thus mediating interactions between the cell wall and the cell’s interior, and therefore ideally suited to transduce CWI changes to the cell (Decreux et al., 2006; Feng et al., 2018). Apart from CrRLK1L proteins, several other proteins belonging to the wall-associated kinase (WAK) and leucine-rich repeat (LRR) receptor kinase families are thought to bind cell wall components and trigger intracellular responses when the cell wall is changed (Herger et al., 2019).

An interesting exception to the kinase-related CWI surveillance portfolio is the proposal that ion channels may be involved in CWI monitoring. Indeed, CWI perturbations may alter cellular turgor pressure, leading to changes in membrane tension and cell wall stresses; two essential parameters triggering the activation of mechano-sensitive channels embedded in the plasma membrane. As a result, ions accumulate in the cell and can trigger downstream processes (Hamann, 2012; Bacete and Hamann, 2020). Hence, primary cell wall composition and integrity need to be tightly monitored and regulated to support cell expansion and to rapidly respond to environmental changes.

In the cell wall, cellulose microfibrils are tethered by the matrix polysaccharides hemicellulose and pectin. Historically, the plant cell wall was described as a macromolecular composite, in which cellulose microfibrils are embedded in a hydrated and complex polysaccharide matrix constituting of hemicellulose and pectins, and in this matrix components were linked to each other in a non-covalent manner, determining cell wall mechanical properties (Cosgrove, 1997). This model was then replaced by a “tethered network” model, in which the role of xyloglucans (i.e. hemicellulose) was revisited and extended as a key structural determinant for cell wall extensibility (Park and Cosgrove, 2015). Structural assessments of the cell wall, using solid-state nuclear magnetic resonance (ssNMR) combined with biomechanical approaches, revealed that pectins cover large surface areas of cellulose microfibril, while xyloglucans rather connect cellulose microfibrils at distinct positions, referred to as “hot spots” (Park and Cosgrove, 2014). These biomechanical hot spots might be targeted by cell wall loosening proteins, resulting in the sliding of cellulose microfibrils along each other, which in turn impacts cell wall extensibility (Park and Cosgrove, 2014). However, the cellulose microfibril sites to make these structures and how such formation is regulated are still not clarified. A possible scenario may involve targeted secretion of matrix wall polysaccharides and subsequent differential diffusion of them. From a biomechanical point of view, the relative impact of the hot spots on cell wall mechanical properties is also not clear, though recent modeling approaches might provide avenues to better appreciate the impact of those (Zhang et al., 2021).

Several studies have touched upon the role of pectins in cell wall mechanics and thus indirectly in CWI (Palin and Geitmann, 2012; Braybrook and Peaucelle, 2013). Pectins are galacturonic acid (GalA)-enriched complex polymers that form gel-like configurations, and thus connect multiple cell wall components (Palin and Geitmann, 2012). Pectins support CWI by directly affecting cell wall porosity and hydration, and probably also the accessibility of wall-modifying enzymes to their substrates (Willats et al., 2001). Homogalacturonans (HGs) are arguably the most common component of pectin and are made up of linear chains of negatively charged galacturonic residues. These residues can be methyl esterified, which regulates their ability to adopt gel-like configurations upon addition of gelling agents, such as Ca^2+^. Indeed, HGs exist in two different forms in the cell wall, and in their secretory route to the apoplast; a largely methylesterified form or a largely de-methyl-esterified form.

These two HG configurations change cell wall mechanics and CWI. In brief, de-methylesterification of HGs, through the action of pectin methylestereases (PMEs), can result in softer cell walls, as HGs become the target of HG-modifying enzymes (HGMEs), such as pectate lyases, or in stiffer cell walls, as de-methyl-esterifed pectins can cross-link to each other via calcium. These two somewhat opposing effects are both supported by experimental data in different tissues (i.e. hypocotyl and pollen tube) (Peaucelle et al., 2015; Bidhendi and Geitmann, 2016). For instance, patterned de-methylesterification of pectins correlates with the mechanical polarity observed in hypocotyl cells during anisotropic expansion (with softer de-methylesterifed longitudinal walls) (Peaucelle et al., 2015). However, the apical ends of pollen tubes contain highly methyl-esterified pectins, which correspond to softening of the walls (Parre and Geitmann, 2005). It is thought that different modes of action of HGMEs could explain these contradictory wall outcomes, hypothetically driven by blockwise (i.e. continuous) or non-blockwise (i.e. discontinuous) demethylation (Bidhendi and Geitmann, 2016). If such enzymatic behavior is demonstrated in vivo, it would be interesting to understand what parameters and components regulate the different pectin patterns.

Cellulose is typically the major component of primary cell walls, and therefore also the most abundant biopolymer on Earth. Cellulose microfibrils are the main load-bearing component of primary cell walls, tethered by polysaccharides, including hemicelluloses and pectins, and largely support directed cell expansion (Baskin, 2005). Contrary to most other cell wall components, which are synthetized in the Golgi apparatus before being secreted to the cell wall through vesicle trafficking, cellulose microfibrils are directly synthesized at the plasma membrane by large protein complexes, referred to as cellulose synthase (CESA) complexes (CSCs) that are organized as six-fold symmetrical rosettes (Polko and Kieber, 2019). The Arabidopsis genome encodes 10 CESA proteins, of which CESA1, CESA3, and CESA6-like proteins (i.e. CESA2, CESA5, CESA6, and CESA9) form heterotrimeric CSCs associated with cellulose production during primary wall formation (Desprez et al., 2007; Persson et al., 2007). The CSCs are estimated to contain 18, or possibly 24 CESAs, with a heterotrimeric ratio of 1:1:1 (Gonneau et al., 2014; Hill et al., 2014). Genetic studies have shown that mutations of several of these CESAs impair cellulose synthesis and result in severe growth phenotypes or gametophytic lethality (Desprez et al., 2007; Persson et al., 2007; Fagard et al., 2000).

While the heterotrimeric CESAs form the catalytic core of the CSCs, it is clear that several other proteins directly associate with the CSCs or contribute to the synthesis of cellulose (for a review, see Polko and Kieber, 2019). Before going into further details on these proteins, it is important to outline the cycle of a typical CSC. As a classic membrane-spanning plasma membrane protein, the CESAs are made at the ER and then assembled into CSCs in the ER or during their route through the Golgi apparatus. The CSCs are then secreted to the plasma membrane through the trans-Golgi network (TGN) and delivered at sites that often coincide with cortical microtubules (McFarlane et al., 2014). Once delivered, the CSCs are activated and proceed to produce cellulose, a process guided by cortical microtubules. When the synthesis is completed, the CESAs become internalized via endocytosis and are then thought to either recycle back to the plasma membrane via the TGN or be degraded (McFarlane et al., 2014). Many of the mechanistic details that support these processes are not well resolved, but the following section gives a brief overview of some of the proteins that assist in different steps of the CSC cycle.

While there are many proteins associated with the different steps explained above, here we outline those that are closely associated with CSC function and regulation. The assembly of CSCs appears to be aided by STELLO (STL)1 and 2 in the Golgi (Zhang et al., 2016b). STLs are proteins consisting of a domain of unknown function (DUF) 288 and perhaps are required for glycosylating some molecules; however, the substrates and targets of such activity are not known (Zhang et al., 2016b). Secretion of the CSCs is regulated by multiple factors, including the status of the actin cytoskeleton (Sampathkumar et al., 2013; Zhang et al., 2019), pH of the endomembrane system (Luo et al., 2016), the exocyst complex and PATROL1 (Zhu et al., 2018), two proteins of unknown function referred to as SHOU4 and SHOU4-like (Polko et al., 2018), and another protein of unknown function named TRANVIA (Vellosillo et al., 2021). Delivery of the CSCs to the plasma membrane is coordinated by cortical microtubules (Gutierrez et al., 2009), but the mechanism behind this coordination is again not well understood.

Once delivered, the CSCs are thought to be activated, possibly via post-translational mechanisms, and cellulose synthesis thus commences. As the stiff cellulose microfibrils get incorporated into the cell wall, they become immobilized and further synthesis therefore causes the CSCs to move forward in the membrane (Paredez, 2006). As the speed of this movement (ranging from around 250 to 500 nm/min) should stem from the catalytic activity, it is estimated that each CESA in the CSCs incorporates around five to six glucose units per second into the growing cellulose chains (Paredez et al., 2006). The direction of CSC movement is largely determined by the orientation of cortical microtubules (Paredez et al., 2006). This mechanism is supported by POM2/CESA INTERACTING (CSI)1 proteins, which physically link microtubules to CESAs at the plasma membrane (Bringmann et al., 2012; Li et al., 2012). Lesions in POM2/CSI1 drastically affect CESA dynamics and reduce the co-localization of CESA trajectories and cortical microtubules, confirming the critical role of this protein in microtubule-based guidance of the CSCs (Li et al., 2012). Other proteins that contribute to this process are the CELLULOSE MICROTUBULE UNCOUPLING (CMU) and the COMPANION OF CESA (CC) proteins (Endler et al., 2015; Liu et al., 2016; Kesten et al., 2019). While many CSCs are guided by microtubules, a recent report highlighted that newly synthesized cellulose microfibrils may also guide the direction of CSCs (Chan and Coen, 2020).

When the primary wall cellulose microfibrils reach a certain length, which should be in the range of 1.75–5 µm based on life-time estimates of the CSCs as around 7–10 min (Sampathkumar et al., 2013), the CSCs become inactive, stall, and are internalized. How the cell perceives the moment when a cellulose microfibril has reached the correct length and the regulatory framework for CSC internalization are unknown, but the CSCs are internalized through clathrin-mediated endocytosis, which involves several proteins, such as ADAPTOR PROTEIN (AP)2, T-PLATE, and TML (Bashline et al., 2013, 2015; Sánchez-Rodríguez et al., 2018). Once internalized, CSCs can be recycled via the TGN or delivered to the lytic vacuole for degradation. Interestingly, whereas the main bulk of fluorescently labeled CESAs appears in the Golgi and plasma membrane, physical perturbation of cell walls by environmental stress or chemical treatment of plants with cellulose synthesis inhibitors, such as the herbicide isoxaben, triggers the trafficking of CSC to cytosolic vesicles, referred to as microtubule-associated cellulose synthase compartments (MASCs) and/or small CESA compartments (SmaCCs), which might act as CSC reservoirs (Crowell et al., 2010; Endler et al., 2015; Lei et al., 2015; McFarlane et al., 2021).

## How do plants perceive and respond to salts?

Plants are thought to sense salinity at multiple cellular sites, including the cell wall, plasma membrane, and intracellular organelles (Zhu, 2016). Here, we briefly summarize some aspects of salt stress sensing and refer the reader to other reviews on this topic that cover these aspects in more depth (e.g. Yang and Guo, 2018; Zhao et al., 2021).

**Figure 1:**
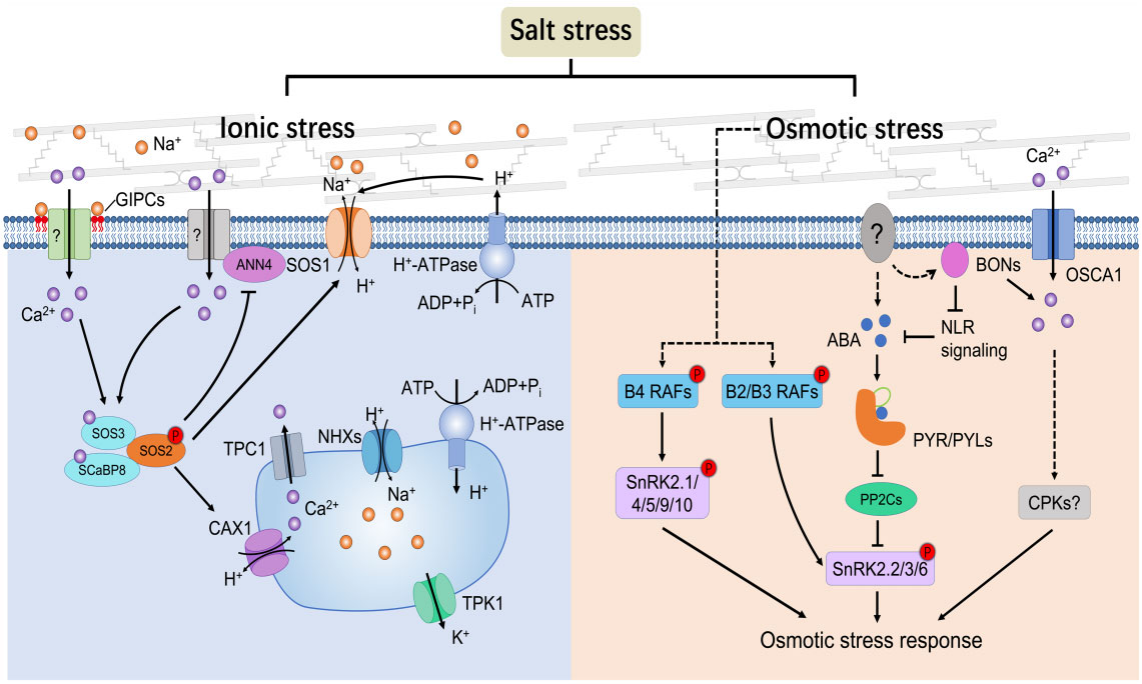
Molecular responses to ionic and osmotic aspects of salt stress. Upon exposure to high salinity, plants suffer both ionic and osmotic stress, the signals of which are thought to be sensed and transduced via distinct pathways. For ionic stress signaling (left panel), apoplastic Na^+^ binds to GIPC sphingolipids in the plasma membrane and triggers Ca^2+^ influx via a yet unknown mechanism. Elevated cytosolic Ca^2+^ activates SOS signaling pathway and promotes Na^+^ extrusion out of cells, driven by the proton gradient created by the plasma membrane H^+^-ATPases. The NHX Na^+^/H^+^ exchangers and vacuolar H^+^-ATPase regulate vacuolar Na^+^ partitioning under saline conditions. Plants may sense hyperosmotic stress through multiple pathways (right panel). For example, the mechanosensitive channel OSCA1 may sense membrane tension changes and start Ca^2+^ influx. The C2 domain-containing BONZAI1 (BON) proteins respond to turgor reduction, regulate Ca^2+^ signals, and repress NLR signaling which antagonizes ABA signaling. Besides, hyperosmotic stress also activates RAF-SnRK2 phosphorylation cascades, and controls stress responses together with ABA and Ca^2+^ signaling.

Salt stress triggers changes to a range of processes at the plasma membrane, such as lipid components, and consequently membrane tension, which might be sensed at the plasma membrane. For example, plasma membrane-based glycosyl inositol phosphorylceramide (GIPC) sphingolipids may bind Na^+^ (and other divalent cations) and are required for salt-triggered Ca^2+^ flux into the cell (Figure 1). However, the molecular mechanisms that drive the elevation of cytosolic Ca^2+^ by GIPCs are unknown (Jiang et al., 2019). Following cytosolic Ca^2+^ elevations, the Ca^2+^ signal is decoded and transduced to trigger intracellular stress responses.

In this context, the salt overly sensitive (SOS) pathway is the best characterized signaling pathway (Yang and Guo, 2018). The SOS pathway consists of the calcineurin B-like protein SOS3 (CBL4), the CBL-interacting protein kinase SOS2 (CIPK24), and the Na^+^/H^+^ antiporter SOS1 (Figure 1). Upon salt stress-triggered increase of cytosolic Ca^2+^, Ca^2+^-SOS3 binds to and activates SOS2, which in turn phosphorylates SOS1 to trigger the extrusion of Na^+^ from the cell’s interior (Halfter et al., 2000). Interestingly, the Arabidopsis *monocation-induced [Ca^2+^]_i_ increases 1 (moca1*) mutant, which is defective in GIPC biosynthesis, phenocopies *sos* mutant phenotypes. Indeed, the GIPC-mediated Ca^2+^ influx is required for the activation of SOS1, further verifying the association of Ca^2+^ signals with the SOS pathway (Jiang et al., 2019).

Other CBL and CIPK family proteins, including CBL1, CBL9, CBL10/SCaBP8, CIPK11/PKS5, and CIPK26 also participate in perceiving Ca^2+^ to regulate the salt stress response (Figure 1; Cheong et al., 2003; Pandey et al., 2004; Lin et al., 2009). Vacuole partitioning of Na^+^ is another critical adaptive mechanism that not only reduces Na^+^ toxicity, but also utilizes Na^+^ as an osmolyte, and is mediated by the vacuole Na^+^/H^+^ exchangers driven by the proton gradient created by vacuolar H^+^-ATPases (Figure 1; Yang and Guo, 2018). However, the identity of the vacuolar Na^+^/H^+^ exchangers remains controversial (Apse et al., 2003; Bassil et al., 2011; Barragán et al., 2012). In addition, the H^+^/Ca^2+^ antiporter CATION EXCHANGER1 (CAX1), as well as the vacuolar two-pore K^+^ channel 1 are required for the regulation of vacuolar ion homeostasis during salt stress (Figure 1; Cheng et al., 2004; Batelli et al., 2007).

Salt stress causes osmotic stress, but how plants perceive the osmotic stress is unclear. Osmotic stress is a physical stimulus that is likely not actively perceived by cells. Instead, plant cells may sense hyperosmotic stress through multiple stress-induced biophysical changes, including turgor reduction, CWI perturbation, cell membrane tension, macromolecular crowding, and cell damage (Nongpiur et al., 2020; Rui and Dinneny, 2020). Nevertheless, some of these changes may be monitored through sensors, which in turn trigger multi-level biochemical changes, including protein modifications and relocalization (Grison et al., 2019), nanodomain formation (Smokvarska et al., 2020), elevation of second messengers (Knight et al., 1997), activation of protein kinases (Chen et al., 2021), accumulation of hormones, and changes in gene expression. These biochemical changes may trigger multiple outputs, such as stomatal movement, metabolic and osmotic adjustment, hydrotropism (Dietrich et al., 2017), growth regulation (Zhang et al., 2020), senescence (Zhao et al., 2016), and dormancy (Tylewicz et al., 2018; Iwasaki, 2022).

While much of the osmotic sensing is, as mentioned above, not well characterized, recent studies have begun to untangle potential mechanisms. For instance, osmotic stress may trigger RLK relocalization, for example, the Arabidopsis plasma membrane-localized LRR receptor-like kinase, QIAN SHOU KINASE (QSK1), is rapidly relocalized to plasmodesmata upon osmotic stress (Grison et al., 2019). However, whether and how QSK1 regulates subsequent osmotic stress responses, such as symplastic cell-to-cell signaling, is still unclear (Mittler et al., 2022). Another mechanism involves the Rho GTPase ROP6 and two NADPH oxidases, RBOHD and RBOHF, which can form stress-triggered nanodomains within the plasma membrane and control apoplastic H_2_O_2_ accumulation (Smokvarska et al., 2020). In animal cells and bacteria, osmotic changes can be detected by membrane tension sensors localized at the plasma membrane (Haswell et al., 2011). A similar mechanism has been proposed in plants, where a mechanosensitive Ca^2+^ channel (OSCA1) might sense membrane tension changes and control cytosolic Ca^2+^ increase (Figure 1; Yuan et al., 2014). More recently, the C2 domain-containing protein BONZAI1 was shown to control Ca^2+^ signals as well as global osmotic stress responses, including abscisic acid (ABA) accumulation, upon osmotic stress (Chen et al., 2020). ABA can be sensed by the pyrabactin resistance (PYR)/PYR 1-like (PYL)/regularly component of ABA receptor (RCAR) family members that interact with and inhibit clade A protein phosphatase 2Cs (PP2Cs), leading to the activation of ABA-dependent SnRK2s, including SnRK2.2/3/6/7/8 (Ma et al., 2009; Park et al., 2010). Although it is still unclear how these early signaling components sense osmotic stress, their plasma membrane localization might indicate complex osmosensory sensing of cell wall and plasma membrane changes. While much of the above sensory systems are located at the cell surface, a slew of downstream signaling cascades are located in the cytosol. One such signal transduction cascade is mediated via RAF-SnRK2 phosphorylation that controls cytoplasmic and P-body activation of SnRK2-mediated processes, which are critical for ABA and osmotic stress responses (Figure 1; Boudsocq et al., 2004; Kobayashi et al., 2005; Saruhashi et al., 2015; Shinozawa et al., 2019; Soma et al., 2020; Takahashi et al., 2020; Lin et al., 2021).

## Salt stress and the cell wall

Salt stress is thought to cause at least three layers of adverse effects on plants, including osmotic stress, ionic stress, and oxidative damage (Van Zelm et al., 2020). In this section, we consider how these interlinked effects impact processes associated with the plant cell wall, moving from the apoplast toward the cortical cytoplasm.

### Apoplast

**Figure 2:**
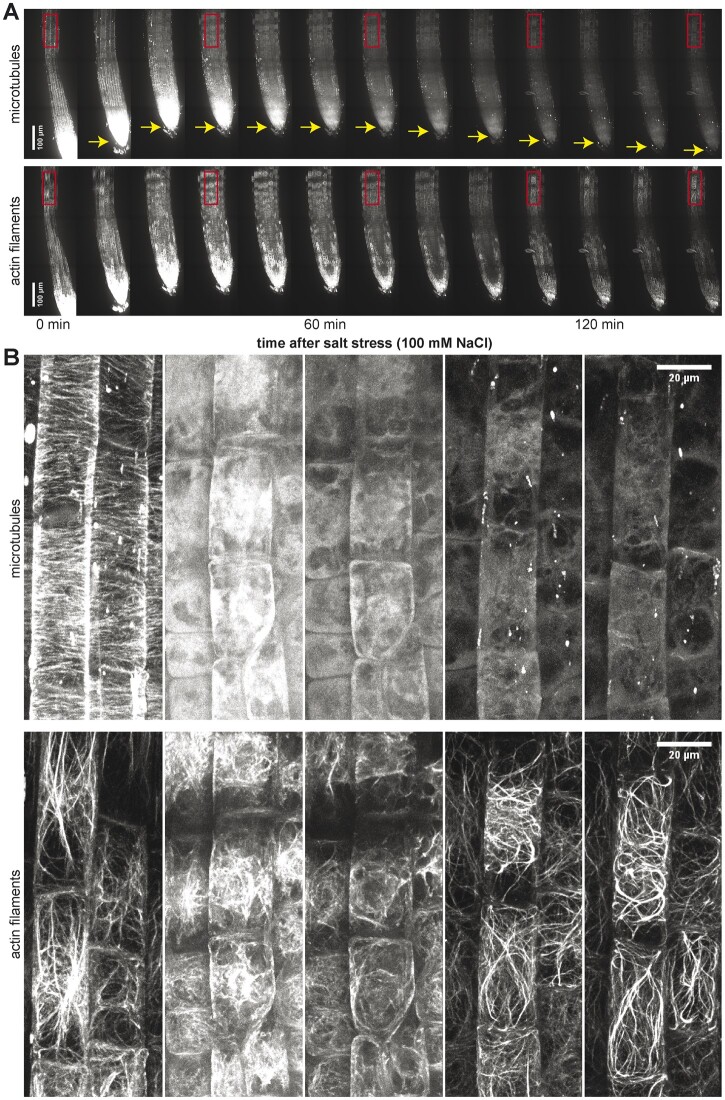
Salt stress depolymerizes plant cytoskeleton in root cells. A, Timelapse of an Arabidopsis root montage after salt treatment (for “Material and methods” see Ruhnow et al., 2022, in press). The upper panel shows microtubule cytoskeleton (p35S:TUA5-mCherry) and the lower panel shows actin cytoskeleton (UBQ10:mNeonGreen-FABD2). Rectangles indicate regions cropped and shown in Panel B. Time after salt stress is indicated below. Arrows indicate root contraction after salt exposure. B, Crops and close-up of regions of the root showing actin (upper panel) and microtubule (lower panel) cytoskeleton depolymerization after salt stress (scale bar = 20 µm). Both actin and microtubule cytoskeletons are depolymerized at 60 min after salt stress. Actin repolymerization is observed after 120 min, while microtubules are still depolymerized.

Osmotic stress is caused by reduced water availability and is the earliest event occurring in plants under saline conditions. Salinity lowers water potential in the soil, resulting in a reduced ability of plant cells to absorb water and reduced cellular turgor pressure, thereby affecting cell expansion. Such changes initially cause a retraction of plant tissue and extrusion of water from the plant cells (Van Zelm et al., 2020). Indeed, the changes in tissue size can readily be observed by following temporal behavior of plant tissues upon salt exposure (Figure 2). To overcome these problems, the cell walls are actively adjusted to adapt to reduced turgor pressure and altered CWI. Within hours or days after salt treatment, and after osmotic adjustment, the osmotic potential gradient between protoplast and apoplast is reestablished to restore tissue size (Figure 2). This will likely be accompanied by cell wall strengthening to resist the adjusted turgor pressure. At this stage, cells in plants with defects in cell wall biosynthesis often burst, which results in severe growth inhibition. For example, Arabidopsis *cesa6* mutant displayed enhanced root cell swelling when grown under high salt conditions (Zhang et al., 2016a). Similarly, a *mur4* mutant, which is disrupted in arabinose biosynthesis, exhibited abnormal root cell morphology and root growth inhibition in response to salt stress (Zhao et al., 2019).

Hence, plants defective in cell wall synthesis and remodeling are typically hypersensitive to salt stress, indicating that CWI maintenance is an important factor for plants to tolerate perturbations in turgor and to reinforce cell walls upon high salinity. As a case in point, mutations of the putative cell wall sensor FERONIA (FER) result in cell bursting during growth recovery under high-salinity conditions, a phenotype linked to defects in cell wall surveillance and repair (Feng et al., 2018). There are several mechanisms that could explain this observation, that is, relating FER to CWI and salt tolerance. For example, the use of fluorescence resonance energy transfer (FRET)-based Ca^2+^ sensors in *fer* seedlings exposed to salt stress revealed reduced transient Ca^2+^ bursts in the root elongation zone when compared with control seedlings. These data suggest that impaired growth recovery during salt stress in *fer* might be linked to Ca^2+^ signaling defects, precluding the activation of downstream responses (Feng et al., 2018).

Another question is how FER detects salt stress-related events in the cell wall. Although it has been proposed that FER might perceive modifications of the cell wall, for example, through its engagement with pectins, there is no clear evidence that such interactions occur in vivo. Another hypothesis was, however, recently raised, suggesting that other proteins might mediate FER interactions with the cell wall. This hypothesis is supported by both co-immunoprecipitation and yeast-two-hybrid assays showing that FER interacts with cell wall-localized LRR extensin (LRX) proteins (Dünser et al., 2019). LRX proteins are cell wall-localized chimeric extensins that can bind to the cell wall via the extensin domain (Herger et al., 2019). Therefore, the FER–LRX interactions could provide a physical link between the plasma membrane (via FER transmembrane domain) and the cell wall (via the extensin domain of LRX) (Dünser et al., 2019), possibly explaining how FER perceives salt stress-triggered cell wall modifications. Consistently, the triple mutations of *LRX3*/*4*/*5* and overexpression of *RAPID ALKALINIZATION FACTOR 22* (*RALF22*), encoding a ligand of FER, both lead to severe salt stress hypersensitivity. This corroborates a role of LRX proteins in FER signaling during salt stress. Because FER is the receptor of RALFs, and LRX proteins can also interact with RALFs, the FER–LRX–RALF connection is thought to form a functional module, acting in a common pathway and connecting salt stress-induced cell wall changes to the regulation of growth and salt stress tolerance (Zhao et al., 2018).

More recently, two other cell wall sensors, the FER-related CrRLK1Ls HERKULES1 (HERK1) and THESEUS1 (THE1), have also been reported to contribute to salt-induced responses. Indeed, the double mutant *herk1 the1-4* (a loss of function allele of *HERK1* combined with a gain-of-function allele of *THE1*) exhibits a similar phenotype to *fer* under salt stress (Gigli-Bisceglia et al., 2022). In this study, the authors proposed a model in which these sensors might mitigate salt stress sensitivity by alleviating salt-induced responses through the negative regulation of MAPK6, a MAPK protein involved in regulating many cellular processes, including salt stress response (Colcombet and Hirt, 2008; Komis et al., 2011). Testing whether these sensors also interact with LRX proteins, or impact cytosolic calcium signaling, would be obvious next steps to place them in context of FER in salt stress signaling.

Salinity causes ionic stress characterized by an excessive accumulation of sodium in roots or shoots, occurring within hours or days after exposure (Roy et al., 2014). Although the cellular targets that are directly affected by sodium are still not fully understood, it is widely accepted that excessive sodium disrupts ion balance, inhibits enzymatic activities, reduces photosynthesis efficiency, and interrupts CWI. Many enzymes participating in primary metabolism require K^+^ to accomplish their activities, but excessive Na^+^ tends to replace K^+^ in those enzymatic reactions and thus reduces metabolic efficiency (Shabala and Cuin, 2008). Therefore, Na^+^/K^+^ ratio is an important indicator that reflects the ability of plants to tolerate high salinity.

**Figure 3:**
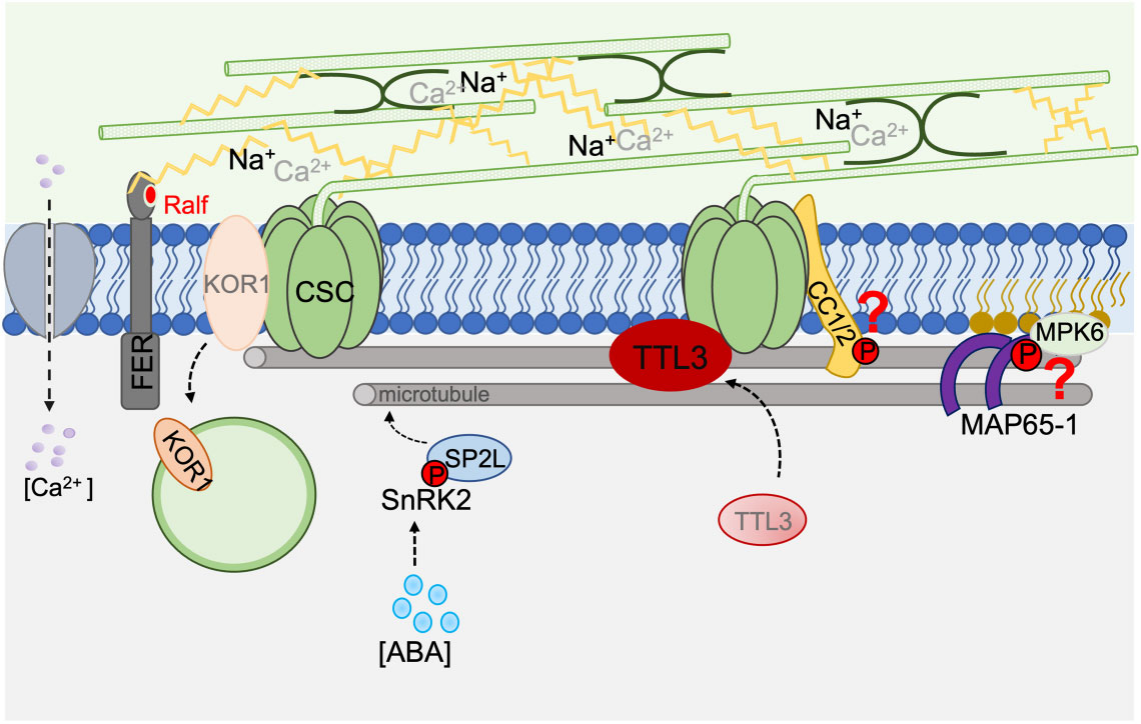
Salt stress and cell wall biology. Accumulation of Na^+^ ions in the cell wall can preclude Ca^2+^ association with pectins perturbing CWI. Wall sensors (e.g. FERONIA [FER]) can detect such perturbations and promote Ca^2+^ uptake by the cell, triggering cell responses. Salt stress also directly impact cellulose synthesis process, notably through the depolymerization of microtubules and CSC (CESA complex) internalization. Several proteins interact and promote microtubule polymerization and bundling during salt stress conditions (e.g. CC1 and CC2, MAP65-1, and MAP SP2L proteins). Recently, localization of other proteins has also been shown to be affected by salinity (e.g. TTL3 and KOR1), where TTL3 contributes to microtubule reemergence reminiscent to that of the CC proteins. Question marks indicate potential phosphorylations that might be triggered by salt stress.

The ionic imbalances that are the result of salt exposure might also directly impact cell wall mechanical properties. Recent AFM measurements have highlighted that salt stress may cause cell wall softening, notably through its effect on pectins (Beauzamy et al., 2015; Feng et al., 2018). Pectins are typically secreted in a highly methyl-esterified form and then selectively de-esterified by pectin methyl esterases (PMEs). Pectins that are de-methylesterified can be cross-linked by divalent cations, such as Ca^2+^, to form egg-box structures and thus promote cell wall stiffness (Hocq et al., 2017). It is thought that excessive Na^+^ in apoplastic region competes with Ca^2+^ to bind to pectin, which in turn disturbs the cross-linking of pectin (Manunza et al., 1998; Figure 3). However, salt stress might also directly trigger PME activation, resulting in a decrease in pectin methylation, thus affecting cell wall properties (Gigli-Bisceglia et al., 2022). Consistently, application of exogenous Ca^2+^, which inhibits PME activity, decreased certain salt-induced responses, furthering the notion that the pectin status is important during salt stress signaling (Gigli-Bisceglia et al., 2022). Salt stress induces GALACTAN SYNTHASE 1 (GALS1) in roots, leading to the accumulation of ß-1,4 galactan, a major component of pectins (Yan et al., 2021), which reduces root growth. The expression of *GALS1* appears to be attenuated by two transcription factors (called BCP1 and BCP2), which then prevents galactan accumulation and thus promotes root growth (Yan et al., 2021). This study provides an interesting example of how a proper balance of cell wall components affects root growth during salt stress. Overall these data highlight the relevance of the pectic composition and divalent ion contributions in salt stress responses.

Abiotic stresses, such as salt, drought, cold, and heat, trigger the production of ROS. Low amounts of ROS may act as a second messenger that initiates stress responses and thus enhances salt tolerance. This notion is supported by genetic data showing that mutations of *AtRbohD* and *AtRbohF* genes, which are required to produce ROS in the apoplast, result in a salt-hypersensitive phenotype (Ma et al., 2012). In addition, salt stress-induced ROS production activates peroxidases involved in cell wall remodeling (Tenhaken, 2015). Indeed, several studies have shown that peroxidases can crosslink wall glycoproteins, such as extensins, as well as components of the secondary cell wall, such as lignins (MacAdam and Grabber, 2002; Wakabayashi et al., 2012). The formation of crosslinks between wall components suggests cell wall strengthening, which might help the cell to withstand osmotic changes caused by salt stress (Tenhaken, 2015). However, ROS also initiate the formation of radicals that may cleave sugar bonds in plant polysaccharides, and thus, causing cell wall loosening (Schopfer, 2001). Similar to the apparent opposing outcomes of pectin esterification above, the ROS-related effects may act in conjunction with a range of other cell wall-related activities and should therefore take into account peroxidase activity, ROS production, and environmental conditions. For instance, substrate availability may determine the outcome of radical formation and thus cell wall loosening (Tenhaken, 2015).

While salinity triggers ROS production, over-accumulation of ROS can also cause further oxidative stress, which can be detrimental to plant survival. Several genetic studies have reported that plants with elevated salt-induced ROS accumulation exhibit leaf bleaching phenotypes. For example, mutation of *FER* leads to constitutively increased accumulation of ROS and reduced survival during salt stress, whereas application of dimethylthiourea, a H_2_O_2_ scavenger, prevented leaf bleaching phenotype of *fer* mutant under salt stress (Zhao et al., 2021a; Figure 3). Disruption of nicotinamide adenine dinucleotide (NAD) biosynthesis via mutation of *QUINOLINATE SYNTHASE* (*QS*) also leads to enhanced leaf bleaching under salt stress, which was associated with excessive accumulation of ROS (Hong et al., 2020; Wei et al., 2020). Overall, these data indicate that tight regulation of ROS homeostasis is critical for plant survival under salt stress.

### Cell surface and plasma membrane

Impaired anisotropic growth is one of the responses of plants grown on high salinity. Since the orientation of cellulose microfibrils largely supports anisotropic growth, several studies have investigated how salt stress impacts cellulose biosynthesis. When exposed to salt, both the CSCs and microtubule cytoskeleton dynamics are perturbed in Arabidopsis cells. Indeed, salt and osmotic stress cause the removal of CESAs from the plasma membrane and increased accumulation of SmaCCs/MASCs (Crowell et al., 2009; Gutierrez et al., 2009), as well as temporary microtubule depolymerization (i.e. 30 min after the exposure) (Komis et al., 2002; Wang et al., 2007). Interestingly, after several hours in saline conditions, microtubules re-assemble, and CESAs re-populate the plasma membrane (Wang et al., 2007; Endler et al., 2015), highlighting that plants have developed strategies to sustain cellulose synthesis under salt stress.

Evidence of such strategies has been provided by the characterization of CC1 and CC2, which are part of the plasma membrane-based CSCs (Figure 3). In the absence of functional CC1 and 2 proteins, growth of etiolated seedlings was impaired when grown on media containing NaCl, whereas growth was not affected when grown on media containing sorbitol, indicating that the response was not due to osmotic stress. In addition, the CC proteins were necessary to recover cortical microtubules and CSC location during salt stress (Endler et al., 2015). CC1 contains four motifs that can interact with microtubules in vitro, promoting either microtubule polymerization or bundling, and enhancing microtubule re-assembly after salt stress (Kesten et al., 2019). Certain modifications of the sequence of these motifs prevented CC1 binding to microtubules, and impaired salt stress tolerance in etiolated seedlings (Kesten et al., 2019), indicating that the interaction between CC1 and microtubules is critical for salt stress tolerance. However, how salt stress modulates microtubule–CC1 interaction remains to be explored.

Notably, sequence analysis of the four microtubule-binding motifs of CC1 revealed certain similarities between them and motifs involved in microtubule-binding in Tau (Kesten et al., 2019). Tau is a microtubule-associated protein (MAP) in animal cells that promotes microtubule stabilization in neurons (Kadavath et al., 2015). Exposure to chronic stresses enhances the risk of stress-related modifications of Tau, and there is evidence that these modifications are related to certain brain-related pathologies (Congdon and Sigurdsson, 2018). For instance, hyperphosphorylation of Tau can affect its interaction with microtubules (Lindwall and Cole, 1984). There is no clear Tau homolog in plants, but given the similarities in microtubule-binding between Tau and CC1, one could speculate that salt stress could also trigger CC1 modifications, changing CC1 behavior in the cell, and ultimately its interaction with microtubules or other cellular components.

Interestingly, CC1 displays multiple possible phosphorylation sites close to their microtubule-binding motifs, and phosphoproteomic database searches revealed that many of these sites can be phosphorylated (PhosPhat database, https://phosphat.uni-hohenheim.de). Whether salt stress triggers or changes CC1 phosphorylation is not clear from these database searches, but could certainly provide a neat mechanism regulating for protein function. It is here worth noting that microtubules have slightly negatively charged surfaces and a phosphorylation of CC1 could therefore mediate a reduction in interaction strength based on repulsion of the negative charges of the phosphogroups and microtubules.

Another CSC component that is also relevant during salt stress is KORRIGAN1 (KOR1; Figure 3). KOR1 is associated with the CSC and has endo-glucanase activity, which presumably acts on the cellulose microfibrils to support cellulose synthesis. Imaging of root cells after salt treatment showed KOR1 internalization (Nagashima et al., 2020), presumably together with the CESAs and other CSC components, such as the CC proteins. KOR1 internalization has been suggested to aid in salt stress tolerance, though it is not clear how (Nagashima et al., 2020). The Nagashima et al. (2020) study was conducted using drug treatments, such as phenylarsine oxide (PAO) or 1-butanol (inhibitor of phospholipase D (PLD)), which indeed changed the KOR1 localization. However, this conclusion should be taken with caution, since these drugs do not act only on KOR1-associated trafficking. For example, PAO and 1-butanol indirectly affect the levels of phosphatidylinositol 4-phosphate/phosphatidylinositiol 4,5-bisphosphate and phosphatidic acid (PA), which act as secondary messengers during signaling and that are important regulators for membrane-associated processes, including cellular trafficking, hormone signaling, and stress responses (McLoughLin et al., 2013; Gujas et al., 2017; Wang et al., 2019). Hence, while the trafficking of KOR1 might be associated with salt stress responses, more specific studies of this trafficking are needed.

Another caveat using inhibitors is the assumption of specificity. For instance, 1-butanol was used to show that PLD may be connected to microtubules, using 2-butanol as control treatment (Gardiner et al., 2003). However, the specificity of 1-butanol for PLD has not been shown in plant biology. Nevertheless, 1-butanol promotes phosphatidylalcohol production instead of PA from PLD, which leads to a significant reduction of PA content at the plasma membrane, and it has been shown that PA is associated with salt stress tolerance (Zhang et al., 2012). Consistently, salt stress sensitivity has been reported for a *pld* mutant that displays less plasma membrane-associated PA. Based on these data, one might expect to observe that KOR1 is mis-localized in this mutant, which perhaps then links back to PLD activity, membrane modifications, and salt stress sensitivity.

### Cell cortex and cytosol

Cell wall, and in particular cellulose synthesis is closely related to the organization of the cortical microtubule array. As indicated above, salt stress causes depolymerization of microtubules (Wang et al., 2007; Zhang et al., 2012). Application of inhibitors, such as oryzalin or taxol that perturb microtubule dynamics, affects plant tolerance to salt stress (Wang et al., 2007). Microtubule behavior is largely regulated by MAPs, which regulate microtubule dynamics and organization (Hamada, 2014). While we outlined the action of CC proteins above, that is, proteins that are directly connected to the CSCs and engage with the microtubules, a range of other MAPs impact microtubule stability. For example, MAP65-1 promotes microtubule bundling both in vitro and in vivo (i.e. in protoplasts) (Figure 3) and thus enhances their stability, and *map65-1* mutants are more sensitive to salt stress (Zhang et al., 2012). Interestingly, MAP65-1 binds to PA, a phospholipid derived from PLD activity (see above). Notably, PLDα1 increases MAP65-1 activity and thus promotes microtubule stability under salt stress conditions (Zhang et al., 2012). In planta, PA and MAP65-1 interact with the mitogen-activated protein kinase 6 (MPK6) (Zhou et al., 2017; Figure 3). *mpk6* mutants exhibit reduced microtubule depolymerization in pavement cells and are hypersensitive to salt stress (Zhou et al., 2017). One hypothesis would therefore be that MPK6 phosphorylates MAP65-1 under salt stress conditions, and then impacts microtubule bundling and stability to better withstand salt stress. In addition, PA can activate MPK6, which then phosphorylates SOS1, during salt stress (Yu et al., 2010). These results indicate that MPK6 might coordinate salt stress responses through different pathways, that is, via both SOS pathway and microtubule maintenance.

Although further studies are required to understand the role of MPK6 during salt stress, these results highlight that microtubule depolymerization (i.e. as in *mpk6* and *map65-1* mutants) during salt stress clearly affects cell survival (Zhang et al., 2012), possibly caused by changes in cell wall flexibility or strength, which is required to face rapid osmotic changes triggered by salt stress. One could hypothesize that a more stable microtubule array would protect cells from sudden turgor pressure changes. This hypothesis is consistent with recent results from *fer* seedlings treated with oryzalin, in which cells burst more frequently than in non-treated *fer* seedlings (Malivert et al., 2021). Conversely, mutations in the ROP-interactive CRIB motif-containing protein1 (RIC1), which interacts with the microtubule severing protein KATANIN1 (KTN1), increase salt stress resistance (Li et al., 2017). RIC1 is another MAP and is an effector of small GTPases called RHO-OF PLANTS (ROP)2 and ROP6, which indirectly contribute to the regulation of microtubule organization in pavement cells via interactions with KTN1 (Lin et al., 2013). Interestingly, microtubules re-polymerize faster in *ric1* mutants after salt exposure compared with control plants (Li et al., 2017), again corroborating the importance of microtubule re-establishment after salt exposure. While microtubules are prominently associated with cellulose synthesis, they might also contribute to salt stress tolerance through other pathways. For instance, patch-clamp experiments on wall-less Arabidopsis cells revealed that a disorganized microtubule array (either caused through mutation or drug treatment) significantly increased the half-life as well as the relative activity of depolarization-activated calcium channels (Thion et al., 1998). These results provide evidence that microtubule cytoskeleton organization can impact Ca^2+^ ion uptake, which is another critical factor in salt stress response (Manishankar et al., 2018).

Two recent studies expand our repertoire of MAPs associated with salt stress. In the first, tetratricopeptide thioredoxin-like (TTL) proteins are implicated as MAPs that also engage with the CSCs (Kesten et al., 2022; Figure 3). The TTLs, which are normally localized in the cytosol of etiolated hypocotyl epidermal cells, are dynamically recruited to CSCs when those cells are exposed to salt. How salt stress triggers TTL3 recruitment to the PM, and whether TTL proteins mainly sense osmotic or ionic stress, or both, is at this point not clear. TTL3 proteins can bind to microtubules, promoting their stability during salt stress, which is reminiscent to the role of the CC proteins (see above). In agreement with the relevance of TTL3 in salt stress tolerance, *ttl* mutant exhibits reduced root growth, cell swelling, and impaired microtubule recovery and CESA re-localization at the plasma membrane during prolonged salt stress.

Interestingly, TTL proteins also work as scaffolds for brassinosteroid (BR) signaling factors at the plasma membrane, a process mediated by the GSK3-like kinase BR INSENSITIVE 2 (BIN2), which is a negative regulator of BR signaling pathway (Amorim-Silva et al., 2019). BIN2 can also phosphorylate CESA1 to reduce CSC activity (Sánchez-Rodríguez et al., 2017; Figure 3). This phosphorylation occurs at a position that is in close proximity to the mapped binding site of TTL3 to CESA1. Moreover, BIN2 impacts plant recovery after salt stress (Li et al., 2020). BIN2 is proposed to localize at the plasma membrane to inhibit SOS2 activity after salt stress, releasing critical components of the BR signaling pathway to the cytosol, which in turn promote growth. These results indicate that salt stress may increase BIN2 abundance at the plasma membrane where it impacts cellulose-related processes. Further work should determine whether BIN2, and more generally the BR signaling pathway, might contribute to the regulation of TTL-based mechanisms allowing sustained cellulose synthesis during salt stress.

Microtubule organization is also impacted by salt stress. For instance, NaCl-induced isotropic microtubule arrays in tobacco (*Nicotiana tabacum*) BY-2 cells (Dhonukshe et al., 2003), and reorientation of the microtubule array, from a transverse to oblique, has been reported in maize root cells exposed to saline conditions (Blancaflor and Hasenstein, 1995). Notably, a recent study revealed that such changes in the microtubule array sustain root halotropism, a phenomenon that is caused by excess Na^+^ and that explains the ability of roots to grow away from patches of high salt (Yu et al., 2022). This mechanism is supported by a change in anisotropic cell growth, which as outlined above is determined by microtubule re-organization. However, the driving forces behind this re-organization have remained unclear.

Yu et al. (2022) showed that the salt-induced microtubule reorientation in root cells is mediated by the stress hormone ABA, through the phosphorylation of the MAP SPIRAL2 (SPR2)-like (SP2L; Figure 3). When salt concentration increases, ABA accumulates in the root tip, activating SnRK2 kinase, which in turn phosphorylates SP2L protein. Interestingly, SP2L is a close homolog of SPR2, a protein that protects microtubule minus-ends to regulate microtubule severing and lifetime (Yao et al., 2008; Fan et al., 2018; Nakamura et al., 2018). In addition, SPR2, together with the microtubule severing protein KTN1, regulates microtubule reorientation after blue light induction in hypocotyl cells (Lindeboom et al., 2013; Nakamura et al., 2018). While these changes are more dramatic than the reorientation during root halotropism, the ability of the SPR2 and SP2L to induce microtubule reorganization to drive changes to cell growth may indicate similar modes of action. Perhaps a future challenge is to investigate the molecular details of how SPR2 and SP2L interact with microtubules through in vitro assays. Interestingly, SnRK2.10 interacts with PA at the plasma membrane (Testerink et al., 2004); a lipid involved in salt stress response in association with MAP65-1 (see also above). One could therefore ask whether PA regulates and coordinates several salt stress signaling pathways in plant cells, in particular associated with the cortical microtubule array.

As mentioned previously, KTN1 drives microtubule reorientation by severing microtubules (Uyttewaal et al., 2012; Lindeboom et al., 2013; Nakamura et al., 2018). KTN1 may also contribute to microtubule reorganization during salt stress (Yang et al., 2019). Here, plants overexpressing *KTN1* exhibited fragmented microtubules in cotyledon pavement cells upon salt stress, while the opposite was observed in *atktn1*, resulting in longer microtubules compared with wild-type cells. These observations are consistent with the microtubule severing activity of KTN1, which promotes microtubule fragmentation. *KTN1*-overexpressing plants exhibited decreased plant salt stress tolerance, whereas *atktn1* plants exhibited improved salt tolerance during early stages of plant growth, despite the loss of this tolerance at later stages (Yang et al., 2019). This study also highlights questions related to differences in salt stress responses and tolerance in the context of different developmental stages. Indeed, the “contradictory” developmentally related results in *ktn1-4* exemplify that mechanisms behind salt stress tolerance may depend on plant developmental stages.

While the microtubule array and its dynamic reorganization are important for cell wall synthesis and salt stress-related processes, much less is known about the major cytoskeletal component actin in this context. Several studies indicate interactions and mutual benefits of the actin cytoskeleton and microtubules (Petrášek and Schwarzerová, 2009; Sampathkumar et al., 2011, 2013; Zang et al., 2021). The actin cytoskeleton also regulates the distribution of cell wall material, CESA-containing Golgi, as well as the exocytic and endocytic rate of CESAs. Indeed, actin cytoskeleton disruption using latrunculin B alters CESA density and distribution at the plasma membrane and inhibits Golgi motility (Crowell et al., 2009; Gutierrez et al., 2009; Sampathkumar et al., 2013). Although little attention has been paid to the actin cytoskeleton under salt stress conditions, the actin organization is clearly affected in Arabidopsis root cells exposed to salt stress (Figure 2). Interestingly, this is in contrast to rice root cells, where the actin cytoskeleton was largely unaffected during salt stress (Liu et al., 2022). The factors that might drive either the stability of the actin cytoskeleton in rice cells, or the changes observed in Arabidopsis are not known. However, a recent study highlighted that salt stress induces the expression of the actin depolymerizing factor (*ADF1*). In the absence of ADF1, a significant increase in the number of actin bundles was noted, together with a decrease in actin cytoskeleton density, leading to a reduced seedling survival rate (Wang et al., 2021).

## Discussion and perspectives

Salt stress is one of the major environmental stresses encountered by plants as it simultaneously causes ionic toxicity, osmotic stress, and oxidative stress, which directly impact plant growth and thus are responsible for important losses in crop production. Hence, knowledge of the molecular mechanisms behind plant adaptation to salt stress is necessary to guide future approaches and mitigate the impact of salt stress on agricultural productivity. Several new studies (noted above) at the intersection of salt stress and cell wall synthesis have substantially advanced this field; however, many questions remain.

An important area where further exploration is clearly needed is the perception of salt stress, and how such perception results in suitable responses. In the context of cell walls, CWI sensing is certainly a key aspect, not only to detect, but also to initiate adaptive responses to salt stress. Here, a recent study revealed that cell wall perturbations might regulate cortical microtubule reorganization through FER and demethylesterified pectin (Tang et al., 2022). The binding between FER and demethylesterified pectin may form a direct link between the cell wall and plasma membrane (Feng et al., 2018; Duan et al., 2020; Tang et al., 2022). Single-cell ablation, compression, and isoxaben treatment trigger mechanical perturbations and possibly also turgor changes. These physical changes activate ROP6 guanosine triphosphatase (GTPase) via FER. Consequently, defects in FER-mediated ROP6 activation negated stress-induced microtubule reorganization (Tang et al., 2022). Besides FER, its homolog BUDDHA’S PAPER SEAL 1 (BUPS1) senses and/or responds to mechanical changes in pollen tubes and activates ROP1-dependent processes (Zhou et al., 2021). Furthermore, yet another FER homolog, THE1 modulates cell wall stiffness and represses hyperosmotic stress-induced ABA accumulation (Bacete et al., 2022). Hyperosmotic stress reduces turgor, while hypoosmotic stress induces turgor increase and such hypoosmotic conditions might trigger FER, THE1, and BUPS1 activity. While these studies highlight the relevance of FER and its close homologs in CWI sensing, it is noteworthy that the Arabidopsis genome encodes around 400 RLKs (Shiu and Bleecker, 2003), with the majority not associated with any clear functions. One might therefore expect to see many more CWI-related sensing modules emerging in the coming years.

Salinity can induce structural changes in cellulose microfibril organization in the cell wall of sorghum (*Sorghum bicolor*), where cellulose microfibrils were less parallel in root cells exposed to salt stress (Koyro, 1997). One could speculate that this is caused by microtubule defects under salt stress, perhaps due to the reassembly of microtubules in a different configuration when compared with untreated plants. An alternative hypothesis could, however, be that the salt perturbs pectins (e.g. by disturbing cross-linking, etc., as per above) and that the defects, or changes, in the pectins then impact cell wall coherence and therefore also disturb cellulose orientation.

Indeed, there are certainly indications that salt stress alters the cell wall architecture. For instance, salinity induces the alkalization of the apoplast that perturbs the activity of cell wall remodeling proteins (EXPANSINs) and enzymes (e.g. hydrolases) (Cosgrove, 2000; Geilfus et al., 2017). Under physiological conditions, cell wall remodeling may lead to cell wall loosening, which promotes cell wall expansion and thus cell enlargement (Cosgrove, 2000). By increasing apoplastic pH, salt stress may in this manner indirectly inhibit cell expansion through the perturbation of cell wall remodeling activities (Byrt et al., 2018). In this context, overexpression of a rose (*Rosa* × *hybrida*) expansin in Arabidopsis improved salt tolerance, while loss of function of AtEXLA2 increased salt sensitivity (Abuqamar et al., 2013; Lü et al., 2013). Consistently, a recent study conducted in rice showed that overexpression of *OsEXPANSIN7* is associated with improvement of salt tolerance. Indeed, overexpression of *OsEXPANSIN7* induced a decrease of ROS and up-regulation of the sodium transporter SOS1. Altogether, these data suggest that EXPANSINs are not only affected by salinity, but are also part of salt tolerance mechanisms (Jadamba et al., 2020). One may anticipate future contributions in this area related to how an increase in the levels of EXPANSINs counteracts the alkalinization of the apoplast and how changes in EXPANSIN activity are coordinated with other aspects of cell wall remodeling.

How CESA internalization is regulated, and the molecular components behind this behavior are largely unknown. CESA internalization has also been observed in plants growing in short day conditions, that is, under limited conditions such as short day situations in absence of external sugar resources, CSCs are active at the plasma membrane during the day and then internalized during the night (Ivakov et al., 2017). The study concluded that the carbon resources of the plant determine whether cellulose is produced or not (Ivakov et al., 2017). Nonetheless, the internalization observed under these conditions may be similar to those observed during the rapid internalization of the CSCs upon salt stress. Perhaps this is driven through post-translational modifications, such as phosphorylation or ubiquitination.

In addition, several studies have demonstrated that the plant circadian clock is involved in adaptive responses to stress, including osmotic stresses (Hotta et al., 2007; Grundy et al., 2015; Seo and Mas, 2015). Strikingly, plants treated with salt during the day are more sensitive to the stress compared with plants treated during the night, that is, the day-treated plants displayed reduced root growth (Park et al., 2016). In this study, the authors showed that *SOS1* gene expression is regulated by the circadian clock. They found that SOS1 protein accumulated during salt stress, and this accumulation only occurred during certain periods of the diurnal cycle (Park et al., 2017). So, could the SOS1-related pathway be related to the regulation of CESA internalization, or are these processes uncoupled? And, does salt stress cause different effects on plant growth during synthesis of cell wall material and the extension of the cell wall matrix? These are questions that will be important to address.

CESA internalization is mediated by endocytosis, and saline conditions typically promote endocytosis, probably because a decrease of turgor linked to a hyperosmotic shock. As this process is most likely not specific to the CSCs, but rather would affect many cell surface proteins, one could speculate whether those proteins also would show a similar re-emergence at the cell surface as that of the CSCs. Interestingly, PA levels increase during salt stress, and clathrin specifically interacts with this phospholipid under salinity (McLoughLin et al., 2013). As noted above, the PA levels largely depend on PLD activity under saline conditions (Yu et al., 2010). These data highlight that lipid composition of the plasma membrane might be important for the establishment of salt stress-related mechanisms. Yet, how the lipid environment of CSCs at the plasma membrane could affect their activity remains poorly understood. As mentioned above, a recent study demonstrated that salt stress triggers KOR1 internalization, and that PLD inhibitor or PAO treatment (inhibitor of the PI4K) prevents KOR1 internalization, resulting in reduced root growth under salt stress conditions. These results indicate that the phosphatidylinositol-4-phosphate could be important for the recycling of CSC components. Consistently, another study has also shown that PI4K could be important for CESA3 internalization (Fujimoto et al., 2015). Other phosphoinositides, such as the phosphoinositol-4,5-biphosphate (PI-4,5-P_2_), are required for recruiting clathrin coat and promoting endocytosis. PI-4,5-P_2_ levels are increased under salt stress, and plants deficient in enzymes regulating PI-4,5-P_2_ biosynthesis are less tolerant to salt stress (König et al., 2008), indicating that this phospholipid is also involved in the regulation of CSC recycling under salt stress conditions. Overall, these data clearly show that salt stress affects lipid content, but further investigations are required to better understand the impact of this lipid remodeling on CSC regulation.

## Notes

The author responsible for the distribution of materials integral to the findings presented in this article in accordance with the policy described in the Instructions for Authors (https://academic.oup.com/plcell) is: Staffan Persson (Staffan.persson@plen.ku.dk).
